# Missing Links in Antibody Assembly Control

**DOI:** 10.1155/2013/606703

**Published:** 2013-12-31

**Authors:** Tiziana Anelli, Eelco van Anken

**Affiliations:** Division of Genetics and Cell Biology, San Raffaele Scientific Institute, Via Olgettina 60, 20132 Milan, Italy

## Abstract

Fidelity of the humoral immune response requires that quiescent B lymphocytes display membrane bound immunoglobulin M (IgM) on B lymphocytes surface as part of the B cell receptor, whose function is to recognize an antigen. At the same time B lymphocytes should not secrete IgM until recognition of the antigen has occurred. The heavy chains of the secretory IgM have a C-terminal tail with a cysteine instead of a membrane anchor, which serves to covalently link the IgM subunits by disulfide bonds to form “pentamers” or “hexamers.” By virtue of the same cysteine, unassembled secretory IgM subunits are recognized and retained (via mixed disulfide bonds) by members of the protein disulfide isomerase family, in particular ERp44. This so-called “thiol-mediated retention” bars assembly intermediates from prematurely leaving the cell and thereby exerts quality control on the humoral immune response. In this essay we discuss recent findings on how ERp44 governs such assembly control in a pH-dependent manner, shuttling between the cisGolgi and endoplasmic reticulum, and finally on how pERp1/MZB1, possibly as a co-chaperone of GRP94, may help to overrule the thiol-mediated retention in the activated B cell to give way to antibody secretion.

## 1. Introduction 

In the arms race between pathogens and hosts, vertebrates developed a long-range weapon of high precision: the antibody. Antibodies are produced by activated B lymphocytes. Once they are secreted, the antibodies spread throughout the organism to recognize and bind to specific epitopes on pathogens. As such, the pathogens are stigmatized as a target for complement or phagocyte mediated attack and (if all is well) elimination. All weaponry is hazardous, because of the risk that it may “go off” at the wrong moment and aim at the wrong target. Thus, it comes as no surprise that the immune system is reined in by a wealth of safety measures (unfortunately, they are not always fail-proof as the prevalence of autoimmune diseases illustrates). Safety dictates that B lymphocytes should not secrete antibodies, unless there is a confirmed sighting of an antigen, that is, when there is a good match between the antigen and the particular antibody which the B lymphocyte happens to express on its surface. At that moment, the B lymphocyte commits to become a plasma cell, which arguably is one of the most prolific secretory cells in the metazoan kingdom as it releases in bulk the antibody that specifically targets that very antigen. How can it be that the same cell that first obstinately prevents antibodies from leaking out makes a volte-face in a matter of a few days and starts to spit out antibodies by the millions? Here we discuss recent findings that shed new light on this fascinating question.

## 2. The B Cell Receptor (Membrane Bound IgM) versus Secretory IgM

**Figure 1:**
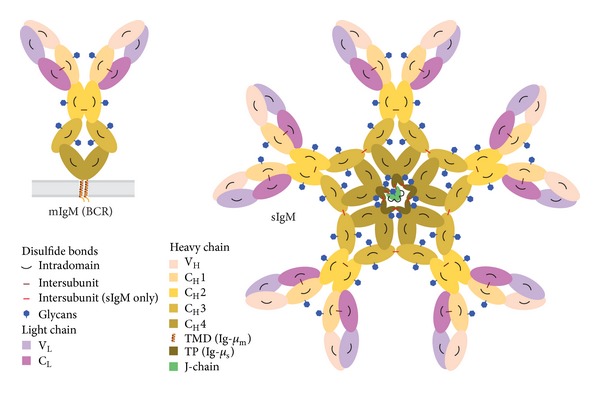
Schematic representation of membrane bound IgM and secretory IgM. B lymphocytes display membrane bound IgM (mIgM)—shown left—on their surface as the core part of the BCR. Once committed to the plasma cell stage they secrete secretory IgM (sIgM) in either “pentameric”—shown right—or “hexameric”—not shown—form. HCs and LCs consist of various Ig-fold domains V_H_, C_H_1–4; V_L_, C_L_ that are color-coded as indicated. Glycans and intra- and intersubunit disulfide bonds between HCs, LCs, and J-chain are depicted. Note that the Ig-*μ*_m_ HC (in mIgM) differs from the Ig-*μ*_s_ HC (in sIgM) in their C-termini, having a transmembrane domain (TMD) or, respectively, a cysteine containing tail piece (TP) as indicated.

To ensure that the antibodies that are secreted recognize the exact same antigen as was originally sighted by or presented to the B lymphocyte, the B cell receptor (BCR) and the antibody are in fact two manifestations of the same molecular device. The core of the BCR is formed by two Ig-*μ* heavy chains (HC) that are covalently linked by a disulfide bond. Each HC is also covalently linked by a disulfide bond to a light chain (LC). Both the HC and the LC are composed of domains that all adopt a so-called Ig-fold, and each of these domains is stabilized by an intradomain disulfide bond. The HCs have one variable domain (V_H_) at the N terminus and four constant domains (referred to as C_H_1–C_H_4 from N to C terminus), while the LCs have next to their N terminal variable domain (V_L_) a single constant domain (C_L_). The C_L_ is juxtaposed to the C_H_1 and the V_L_ to the V_H_ at the tips of the BCR (Figure 1). Together the V_H_ and V_L_ determine the specificity of the BCR in antigen recognition. An ingenious series of recombination events lead to the shuffling of the sequence of the V domains and ensure that the immune system can employ a vast repertoire (estimated at 10^7^ different so-called idiotypes) of Igs, with each B lymphocyte displaying only one particular variant that matches a single particular antigenic epitope. The tetrameric (H_2_L_2_) IgM that forms the core of the BCR is anchored to the plasma membrane via transmembrane domains at the C-terminal part of the Ig-*μ*_m_ HCs downstream of C_H_4 [1].

Secretory IgM consists of nearly identical H_2_L_2_ units (Figure 1). The LCs are indeed the same, but the secretory Ig-*μ*_s_ HCs differ from membrane bound Ig-*μ*_m_ HCs in their C-terminus, since through alternative splicing the last exons have been replaced. In place of a transmembrane domain, the C terminus of Ig-*μ*_s_ HC now displays a hydrophilic tail piece (TP) with one cysteine that is key both for preventing premature secretion and for the assembly of mature secretory IgM (see below). Once it commits to the plasma cell stage, the TP cysteine is used, in fact, to covalently link H_2_L_2_ “monomeric” units in polymeric secretory IgM by disulfide bonds [2]. As a result, plasma cells secrete IgM either as “hexamers” (H_2_L_2_)_6_ or as “pentamers” (H_2_L_2_)_5_J, in which a third antibody component, the J-chain, joins the “monomeric” units, again by disulfide bonds [1]. Altogether secretory IgM consists of entities that each exceeds 1 megadalton in molecular weight, as they are composed in the “pentameric” state of 21 polypeptides, bearing in total 51 N-glycans, and containing 98 intrachain and interchain disulfide bonds [1] (Figure 1).

## 3. Folding and Quality Control of IgM

Secretory proteins and proteins that are displayed on the plasma membrane are synthesized in the endoplasmic reticulum (ER) and the Igs are no exception. The nascent polypeptides obtain glycans and disulfide bonds in the ER as they fold with assistance of the array of ER resident chaperones and oxidoreductases [3]. In fact, the majority of those chaperones and foldases have been implicated in the maturation of antibodies as well as some folding assistants in the intermediate compartment between the ER and Golgi (ERGIC). To name the key players: the “classical” chaperones BiP (originally discovered as being bound to HC in cells lacking LC and hence named Ig binding protein and later found to be the ER resident HSP70) [4] and GRP94 (the ER resident HSP90) [5]; members of the protein disulfide isomerase (PDI) family (PDI itself, ERp72 [6], and–most relevant for this essay–ERp44 [7]; the lectins calnexin [8] and ERGIC-53 [9]; and, most recently, a plasma cell specific ER resident chaperone called pERp1 [10, 11] or MZB1 [12]. The chaperones interact with folding or assembly intermediates recognizing particular regions that are manifest to their immature state. Exposed hydrophobic patches are BiP and/or GRP94 targets; PDI family members recognize aberrant disulfide bonds or free cysteines; finally, calnexin (and calreticulin) recognizes monoglucosylated glycans [13–15]. As such, the nascent proteins are bound to the relay of chaperones until they reach their fully folded mature conformation.

Except for the lectins calnexin and ERGIC-53, the chaperones listed above are all soluble proteins that display a tetrapeptide KDEL (or KDEL-like) sequence at their C terminus. In case any of these chaperones either alone or in complex with a folding intermediate leaves from the folding promoting environment of the ER to stray along the secretory pathway, they will be captured by the KDEL receptors in the ERGIC or cisGolgi. From there the KDEL receptors with their cargo are shuttled back to the ER for further folding attempts [16, 17]. This retrieval system thus is instrumental to quality control of protein folding in the early secretory pathway. Along the same lines, assembly of LCs onto HCs is ensured. BiP binds to the unfolded C_H_1 domain of the HC and facilitates its retention in the early secretory pathway, until LCs manage to displace BiP, and the heteromeric complex is “approved of” by the proximal ER quality control systems [18, 19].

## 4. Assembly Control of Secretory IgM and the Role of ERp44

**Figure 2:**
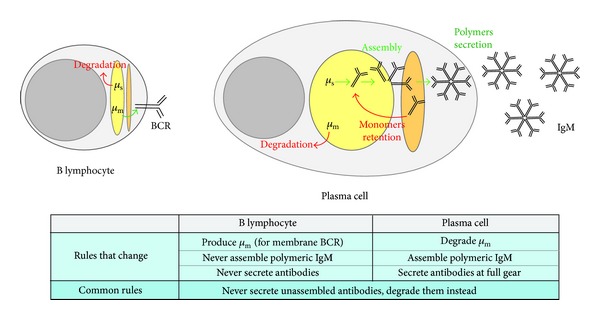
Rules for IgM processing in B lymphocytes and plasma cells.B lymphocytes do not produce secretory antibodies. In B lymphocytes Ig-*μ*_m_ HC (*μ*_m_) is synthesized in the ER; it is assembled in the early secretory pathway (in yellow) and then proceeds through the Golgi (orange) to be exposed on the plasma membrane as part of the BCR; Ig-*μ*_s_ HC (*μ*_s_), instead, is retrotranslocated from the ER and degraded. Conversely, plasma cells are hardworking antibodies factories; they degrade *μ*_m_ but efficiently assemble *μ*_s_ into polymeric IgM and secrete them in bulk. Incompletely assembled IgM subunits are retained in the secretory pathway for another chance of being inserted into a polymer or to be finally degraded.

Central to the effectiveness of the immune response is that unassembled IgM subunit should never be secreted. Indeed, nonpolymeric IgM would bind antigen but fail to fix complement. Thus, the plasma cell must orchestrate that correctly folded and assembled secretory H_2_L_2_ “monomeric” units oligomerize into mature secretory IgM. The task at hand is twofold; on the one hand polymerization must be promoted and polymers must be let go of by the ER quality control mechanisms; on the other hand unassembled “monomers” must be barred from traveling further along the secretory pathway and retrieved for further polymerization attempts (Figure 2).

IgM oligomerization is favored by the hexameric lectin ERGIC-53 [9], which captures fully folded and assembled “monomers” leaving the ER and, probably by organizing them in a planar way, may help the process of polymerization. Correctly assembled IgM polymers presumably then detach from ERGIC-53 in the Golgi and proceed along the secretory pathway. Release of cargo from ERGIC-53 in general seems to be facilitated by the progressively lower [Ca^++^] they encounter traveling from the ER to the Golgi [20]. In the case of IgM, moreover, the polymerization process itself could drive the detachment of the lectin ERGIC-53, because the glycan on the Ig-*μ* TP (Figure 1) that is recognized by ERGIC-53 in the context of IgM “monomers” [21] may become inaccessible in the polymer. Genetic defects in ERGIC-53 or in its partner protein MCFD2 lead to combined deficiency of factor V and factor VIII, since both clotting proteins rely on the ERGIC-53/MCFD2 complex to be actively transported out of the ER and then finally to be secreted into the blood stream [22, 23]. Yet, loss of ERGIC-53 or MCFD function does not result in an apparent impairment of IgM secretion (our unpublished results and [21]); hence ERGIC-53 may be auxiliary to but cannot be essential for polymeric assembly of IgM.

**Figure 3:**
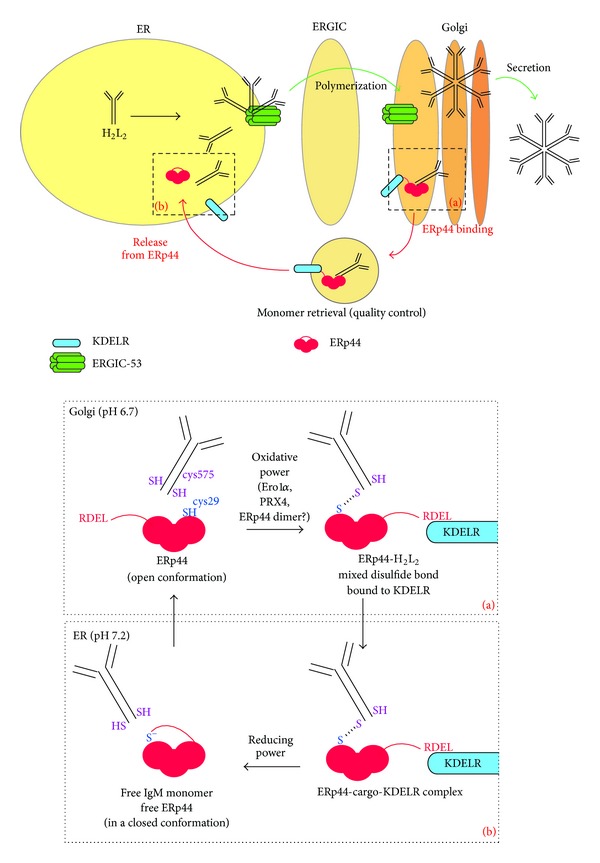
ERp44 quality control and IgM polymerization. IgM monomers are assembled into polymers in the early secretory pathway with the help of the hexameric lectin ERGIC-53. IgM polymers are released from ERGIC-53 in the Golgi and can then be secreted. In the Golgi, ERp44—in an open conformation thanks to the slightly acidic pH—interacts with unassembled IgM subunits exposing the C-terminal cysteine 575 in the SH conformation (inset (a)): the covalent binding between ERp44's active site cysteine 29 and the IgM subunits requires an oxidative power whose source is still unknown. The interaction with the cargo favors the exposure of ERp44's C-terminal RDEL for binding to the KDEL receptor and the complex KDEL receptor-ERp44-cargo (IgM H_2_L_2_ “monomer”) is then transported back to the ER. Here, a reducing power disentangles the disulfide bond between ERp44 and the IgM “monomer.” The neutral pH in the ER, which also inhibits the KDEL receptor activity, now stabilizes ERp44 in a more closed conformation (inset (b)). Keeping ERp44 in a closed conformation in the ER likely is important to avoid that ERp44 interacts with subunits that productively take part in the polymerization process.

Recent findings now also implicate ERp44, an ERGIC-53 partner, in patrolling IgM assembly. In the ERGIC-cisGolgi region, downstream of the “regular” quality control mechanisms in the ER, ERp44 governs a dedicated assembly control cycle for disulfide-linked polymers [9, 24]. ERp44 is a chaperone of the PDI family, composed of three thioredoxin-like domains (a, b, and b′) and a C-terminal tail which ends with an RDEL sequence. The C-terminal tail covers the active cysteine and the surrounding hydrophobic regions in domain a [25, 26]. ERp44 is localized more distally along the secretory pathway than other PDI-like proteins, being enriched in the ERGIC-cisGolgi region [9, 27]. By shuttling from the ER to the Golgi, ERp44 exploits the pH gradient in the early secretory pathway (pH 7.2 in the ER; pH 6.7 in the Golgi) to efficiently block the exit of unassembled subunits with exposed free thiols, which in the mature oligomer will be employed for inter-subunit disulfide bonds [24] (Figure 3).

At the more acidic pH of the Golgi the C-terminal tail opens, unveiling the active site cysteine and the surrounding hydrophobic region for interaction and formation of a mixed disulfide bond with the client proteins. The tail opening also leads to the exposure of the RDEL sequence to the KDEL receptors, which, in turn, allows the KDEL receptor/ERp44/cargo complex to be brought back to the ER (Figure 3, inset (a)). In the ER, instead, the higher pH stabilizes the tail in the closed conformation, which precludes ERp44 from binding its substrates too early during the quality control process (Figure 3, inset (b)). The pH-driven cycle likely involves protonation of the active site cysteine in the more acidic cisGolgi and deprotonation in the more alkaline ER. In the deprotonated thiolate state, the active site cysteine supports a network of electrostatic interactions that keep the tail associated with the surroundings of the active site and thereby keeps it in the closed conformation. In the protonated state these interactions are weakened such that the tail dissociates and ERp44 adopts an open conformation [24].

Thus, ERp44 is central to prevent secretion of unassembled immunoglobulin IgM subunits [7, 9]. Upon retrieval to the ER, the intermediates get another chance of being correctly assembled or—if the assembly attempts are ultimately unsuccessful—become degraded [28]. ERp44 activity not only concerns retrieval of IgM assembly intermediates, but likely patrols disulfide-linked assembly in general. For instance, ERp44 also retrieves unassembled adiponectin from the cisGolgi to the ER in adipocytes [29, 30] and exerts quality control on proper disulfide bond formation of the serotonin transporter protein (SERT) [31]. Moreover, ERp44 is responsible for keeping some partner proteins retained at their appropriate intracellular location in the early secretory pathway such as Ero1*α* [7, 32], SUMF1 [33], and peroxiredoxin 4 (PRX4) [34].

## 5. Missing Links in the IgM Assembly Model

The pH regulation of ERp44 may explain how the assembly process of IgM is patrolled by virtue of missing (disulfide) links. Yet, despite the elucidation of this elegant assembly control, there are several missing links in our understanding of how B cells finally commit to IgM secretion. For one, little is known about the mechanisms that promote the incorporation of J chains into polymers that as a result become “pentamers” and how the plasma cell balances its “hexamer” and “pentamer” output [35]. Then, several aspects of the ERp44 cycle are still unclear. One question is how clients are let go of in the ER, in particular how the mixed disulfide bond between ERp44 and its client is reduced (Figure 3, inset (b)). Likewise, the oxidative source that allows the formation of mixed disulfide bonds with clients in the cisGolgi remains to be identified (Figure 3, inset (a)). The active sites of the majority of PDI-like proteins have two cysteines that form a disulfide bond, which then is donated to clients. Having only a single active site cysteine, ERp44 cannot deliver the oxidative equivalent for a mixed disulfide with clients. Perhaps, Ero1*α*, ERp44's foremost partner [25], provides the oxidative equivalents, as it is also responsible (in part) for reoxidizing PDI [36, 37]. Another partner of ERp44, PRX4, similarly may play such a role. For instance, ERp44 could form a tandem with Ero1*α* or PRX4 but release these partners once they catalyze formation of mixed disulfide bond of ERp44 with a client protein. Another scenario could be envisaged in which the oxidative equivalents are provided in the form of a disulfide bond between two ERp44 proteins using the free (active site) cysteines. In that case, the clients may displace one of the ERp44 molecules forming a mixed disulfide with the other. The free ERp44 then can team up with another free ERp44, whereupon formation of the disulfide that links the ERp44 homodimer may be catalyzed by PDI, Ero1*α*, or the like. Indeed, a substantial fraction of ERp44 is present in the cell as a disulfide linked dimer [7].

Arguably the most intriguing missing link in our appreciation of IgM secretion control is what eventually will overrule the thiol-mediated retention. The unstimulated B lymphocyte already makes some Ig-*μ*_s_ HC but secretes little if any antibody (Figure 2). Instead, all Ig-*μ*_s_ HCs are retrotranslocated into the cytosol to be degraded by the proteasome [28, 38]. Apparently, there is a switch from a nonassembly competent to an assembly competent state in the course of the B to plasma cell differentiation process, which in fact involves an overhaul of the complete cellular makeup. In the course of a few days, cells first stock up on metabolic prowess and then gradually enlarge the secretory pathway to a most impressive capacity before they actually initiate bulk antibody secretion around two days after activation [39]. The full-blown plasma cell then secretes a record load of IgM, which amounts to the equivalent of its own mass on a per day basis. After a week or less of this massive secretion, however, the majority of plasma cells die, while few will commit to long-term survival to sustain B cell memory [1].

Dormant B lymphocytes are not exceptional in being barred from secreting IgM, since also other cells that are transfected with constructs for all secretory IgM components fail to efficiently secrete it [9, 21]. Instead, plasma cells are exclusive in becoming efficient antibody secretors. Thus, the key to the “assembly switch” must lie in the reprogramming of the B lymphocyte in the first days of the differentiation process. One could argue that promoting polymerization simply involves mitigating ERp44 activity. Yet, the retention of unassembled IgM is unabated in plasma cells, as only fully assembled IgM polymers are secreted. In accordance, ERp44 is upregulated in the course of B cell differentiation coordinately with all other folding factors in the early secretory pathway, including ERGIC-53, Ero1*α*, and PRX4 [9, 39, 40]. Moreover, redox conditions in the ER do not seem to alter dramatically during differentiation, as the glutathione GSH/GSSG balance essentially remains the same [41]. Likewise, several proteins involved in redox homeostasis increase during B cell differentiation, but they do so commensurate with the expansion of the secretory machinery, apparently to match the growing need for disulfide bond formation [39, 40].

## 6. pERp1/MZB1: An “Assembly Switch”?

One candidate that may embody the “assembly switch” is pERp1 [10, 11] also known as MZB1 [12]. This protein is exclusively expressed in the ER of B cells and is highly upregulated in the course of the differentiation process to reach levels that are equivalent to the most abundantly expressed ER proteins, such as BiP and GRP94 [11]. Its expression levels are the highest in marginal zone B cells [12], in accordance with their being the most prolific IgM secretors [42]. Importantly, knockdown of pERp1/MZB1 slows down IgM polymerization and thus leads to reduced IgM secretion [11, 12].

Further support for the notion that pERp1/MZB1 is key for IgM assembly comes from a recent study on Kaposi-associated herpesvirus (KSHV). Herpesviridae are notorious for their misleading of the immune system. Upholding herpes family values, an estimated one-third of the KSHV genome indeed is dedicated to immune evasion [43], and as it turns out, this includes the K4.2 immediate early gene. Namely, the K4.2 gene product associates with pERp1/MZB1 and thereby somehow sabotages its function, resulting in a markedly reduced efficiency of IgM secretion from plasma cells [44].

As opposed to KSHV driven shipwrecking of pERp1/MZB1, another pathologic condition involves uncontrolled pERp1/MZB1 expression. MicroRNAs (miRs) contribute to the regulation of gene expression through annealing with target mRNAs, causing their degradation or translational inhibition. A recent report now implicates pERp1/MZB1 as the most prominent target of miR-185 [45], while it had been shown already that reduced levels of miR-185 cause autoantibody production [46, 47]. These findings suggest that miR-185 insufficiency leads to uncontrolled pERp1/MZB1 levels, which in turn gives way to unchecked IgM polymerization and hence unwarranted secretion of antibodies, including autoantibodies. This miR-185 is haploinsufficient in the vast majority of individuals with a ~3 ∗10^6^ bp deletion in chromosome 22 at the q11.2 location. This 22q11.2 deletion syndrome is a relatively prevalent (1 in 2000–4000 live births) genetic disorder that can lead to a wide range of symptoms including congenital heart disease, renal and/or skeletal abnormalities, and learning difficulties, owing to the haploidy of the ~30–40 genes in the affected region [48]. Importantly, symptoms of the 22q11.2 deletion syndrome include an increased prevalence of autoimmune disorders and B cell defects [47] that may well stem (at least in part) from lowered miR-185 levels and concomitant uncontrolled pERp1/MZB1 function.

All these findings indeed point at pERp1/MZB1 as key for IgM assembly. Yet, how pERp1/MZB1 would assist IgM polymerization remains unclear. With the idea in mind that thiol-mediated retention should be counteracted, it was explored whether pERp1/MZB1 could fulfill a role as thiolreductase, but evidence for such activity was meager [11] to nonexistent [12]. Therefore, pERp1/MZB1 most likely derives its putative proassembly effect from a nonthiol related activity. Homology searches already revealed that pERp1/MZB1 is not related to any of the known chaperone families [11]. Instead, pERp1 has several distant family members in a variety of organisms ranging from plants to humans [12].

**Figure 4:**
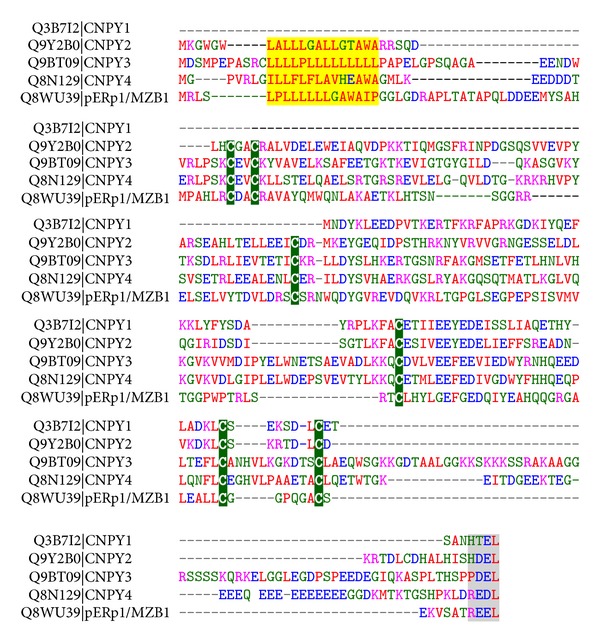
Alignment of pERp1/MZB1 and members of the CNPY protein family. The alignment of the five human protein sequences with UniProt identifiers as indicated was generated with the ClustalW algorithm and manually curated. The signal peptide encoding residues are highlighted in yellow, the C-terminal KDEL-like tetrapeptides are highlighted in grey, and the conserved cysteines in green. Standard color coding for the residues is as follows: alkaline in pink; acidic in blue; other hydrophilic in green; and hydrophobic in red.

After evaluating sequence homologies anew, we now report that pERp1 is part of the saposin-like protein family with, as its closest relatives the canopy homolog proteins CNPY1-4; an alignment is shown in Figure 4. The CNPY proteins and pERp1/MZB1 share a common arrangement of cysteines, indicative of a conserved disulfide bonded structure, an N-terminal signal peptide, and a KDEL-like C-terminal tetrapeptide. Note that the alignment suggests that CNPY1 lacks the N-terminal sequence contained in other family members; however, CNPY-like encoding regions are present in the genomic locus upstream of the annotated CNPY1 initiation (data not shown) and in homologs in other species [49]. Altogether, the shared sequence elements strongly suggest a common residency and related activities in the early secretory pathway for pERp1/MZB1 and the CNPY family members. Along these lines pERp1/MZB1 may well be renamed CNPY5.

Mammalian CNPY1 has not been studied but in zebrafish it associates with the fibroblast growth factor (FGF) receptor. This activity is essential for proper FGF signaling in the developing brain of the fish [49]. CNPY2 was recently reported to enhance expression levels of the LDL receptor in hepatocytes in an FGF21 dependent manner [50]. CNPY3 and 4 were identified as PRAT4A and [51], PRAT4B, respectively [52]. CNPY3 is important for Toll-like receptor (TLR) expression levels [51] except for those of TLR3 [53], while such an effect for CNPY4 has been confirmed for TLR4 only [52].

Several cochaperones of the cytosolic chaperone HSP90 have been identified [54], but for its ER resident paralog, GRP94, the existence of cochaperones for long remained elusive [55]. Excitingly, CNPY3 appears to act as a cochaperone of GRP94 [53]. As such, it assists the folding of TLRs by enhancing or modifying the GRP94 chaperone function, although at present it is unclear at what stage in the folding or dimerization of TLRs the GRP94/CNPY3 tandem comes into play [56]. Altogether it is tempting to speculate that CNPY family members act as cochaperones of GRP94 with CNPY1 assisting FGF receptor folding, CNPY2 LDL receptor maturation, and that pERp1/MZB1 may drive IgM polymerization by virtue of its assistance to or modulation of GRP94 function. In line with this scenario, pERp1/MZB1 interacts with GRP94 [10, 12]. Moreover, pERp1/MZB1 promotes cell surface expression of integrins, which also are GRP94 clients [12]. Finally, it is noteworthy that pERp1/MZB1 interacts with the PDI family member ERp57 as well [12]. Perhaps, the GRP94-pERp1/MZB1 tandem recruits ERp57 to catalyze formation of the IgM inter-subunit disulfide bonds.

## 7. Concluding Remarks

In all Ig secreting vertebrates (from cartilaginous fish onwards) the first wave of the antibody response appears to involve polymeric IgM, whether “tetrameric” (in teleost fish), “pentameric”, or “hexameric” [57]. Polymeric IgM has a higher valency than “monomeric” IgM, which compensates for the low affinity of this first wave antibody response. Another driving force for the primary response to obligatorily involve polymeric IgM may well be the avoidance of leakiness. Premature secretion of antibodies on the one hand can lead to autoimmune effects if B cells happen to produce antibodies that cross-react with self-antigens. On the other hand, they may inadvertently lead to a prophylactic effect by competing with the BCR for scarce antigens and thereby ultimately prevent full scale B cell activation and memory. ERp44 and its rigorous role in thiol-mediated retention seem to effectively preclude the leakiness, but this categorical retention, in turn, necessitated the invention of an escape valve when the B cells become a plasma cell. A tightly controlled switch to polymerization seems to fulfill that role. We argue that pERp1/MZB1, whether in conjunction with GRP94 or not, serves the B cell in switching from a nonsecretory to a secretory phenotype.
